# An ultrafast time-resolution method based on picosecond pulsed laser for determining rock fracture toughness at multipoint during the crack propagation

**DOI:** 10.1038/s41598-022-08428-1

**Published:** 2022-03-16

**Authors:** Mingyuan Zhang, Dejian Li, Liu Yang, Lu Chen, Muao Shen, Junhao Huo, Yingjun Li

**Affiliations:** 1 State Key Laboratory for GeoMechanics and Deep Underground Engineering, China University of Mining and Technology, Beijing, 100083 China; 2 Institute for Deep Underground Science and Engineering, Beijing, 100083 China; 3 School of Mechanics and Civil Engineering, China University of Mining and Technology, Beijing, 100083 China; 4 Institute of Physics, Chinese Academy of Sciences, Beijing, 100190 China

**Keywords:** Civil engineering, Mechanical engineering, Applied optics

## Abstract

An innovative ultrafast time-resolution method based on a picosecond pulsed laser was employed to investigate the mode-I crack propagation characteristics of fractured rock. Its time resolution is as fast as the degree of 45 picoseconds. Then, a series of three-point compressive loading tests with this method were conducted on tuff semi-circular bend (SCB) specimens. Based on this method, we found that the mode-I fracture process of the tuff specimens were composed of repeated crack initiation, arrest, and re-initiation. In addition, the experimental results showed that the fracture rates of the tuff specimens in the initial 10 μs were 636 m/s, 663.9 m/s, and 578 m/s. In comparison, the fracture rates of the specimens were 11.19 m/s, 19.23 m/s, 26.79 m/s during the whole fracture process. As a typical heterogeneous material with primary defects, rock has different fracture toughness at different locations. Therefore, we proposed a new method for determining rock fracture toughness at multipoint during the crack propagation. This new method emphasizes the effect of fracture toughness on crack propagation, which enables to determine the fracture toughness at multipoint and is closer to the original definition of fracture toughness.

## Introduction

Rock as a natural brittle material, the deterioration and structural failure are closely related to its internal tiny cracks. They will expand and propagate when impacted. It is of great help to engineering and the social economy for experimental studying rock fracture. Specifically, it is of great significance to understand the compression between continental plates and better reveal the causes of earthquakes^1,2^. Additionally, it provides a more economical and safe strength theory for slope, surrounding rock support, and tunnel design^3,4^. The critical issue with it is how to describe the whole process of crack initiation, propagation, and arrest^5^. Therefore, it is critical to propose an ultrafast time-resolution method for capturing and describing the whole process of rock fracture.

Griffith reported the first systematic theory of fracture in 1921. He pointed out that strain energy released by fracture must be greater than the surface energy^6^. The main weakness with this theory is that it is only applicable to linear-elastic fracture. Then, Irwin supplemented Griffith's theory, demonstrating that fracture needs to be overcome not only surface energy but also plasticity energy. Furthermore, Irwin proposed the parameter of stress intensity factor based on the concept of strain energy release in 1957. When the fracture occurs, the critical value of the stress intensity factor is called fracture toughness^7^. Fracture toughness is an essential material property independent of specimens’ geometry and external load. It can be considered as the ability of a material to hinder crack propagation. Based on these, the fracture mechanics of brittle materials is developing rapidly. Compared with other brittle materials, rock is heterogeneous, and its internal structure is complex. It means that further experimental study and theory improvement need to be conducted for rock fractures based on traditional fracture mechanics.

In 1965, the first experimental study of rock fracture, by Hoek and Bieniawski^8^, focused on the initiation and propagation of fracture in a biaxial compressive stress field. And the mechanism of rock fracture propagation under biaxial stress was explained. In 1966, Brace used photoelastic material samples to investigate rock fracture and proposed a two-dimensional crack slip cracking model^9^. He explained expansion before the rock failure under the compression through this model. Then, Lajtal used fractured gypsum and granite specimens to examine the initiation and propagation of microfractures and their contribution to material failure in compression in 1974^10^. He discussed the later stages of fracture in terms of a modified Coulomb model and divided the process of brittle fracture in compression into six stages. Several years later, Horri and Nemat-Nasser proposed a two-dimensional mathematical model to analyze the brittle-ductile transition process. In addition, a closed-form analytic solution is presented in this literature^11^. Cao P. et al. investigated fracture coalescence by loading rock-like specimens with two and three pre-existing flaws. And seven types of coalescence had been identified based on their experimental results^12^. Some researchers studied the fracture and failure of rocks from a microscopic point of view. A microscopically-based model of brittle-elastic behavior of compressed rock was constructed by Kachanov to explain the Macroscopic stress-inelastic strain relations in 1982^13,14^. Fanella and Krajcinovic developed a general three-dimensional micromechanical constitutive theory for plain concrete subjected to uniaxial and triaxial compressive loads^15^. With the development of computer techniques and numerical methods, the researchers analyzed the experimental results by combining numerical calculation with laboratory experiments. In 2016, Cao R. et al. investigated by combining similar material testing and numerical simulation using the two-dimensional particle flow code and classified failure patterns into four categories^16^. Lin Q. et al. analyzed jointed rock mass containing a circular hole specimen under compression-shear loading by DIC and DEM modeling^17^. Wu T. et al. investigated the effect of different horizontal distances between the centroids of holes and fissures on the mechanical properties of pre-flawed rock-like material by AE, DIC, and two-dimensional Particle flow code (PFC^2D^)^18^. Lin Q. et al. investigated the mechanical characteristics of a jointed rock mass with double circular holes under uniaxial loading by discrete element method^19^. Previous studies mainly focused on the causes and influencing factors of crack initiation and propagation. However, relatively little work has been done to describe the specific propagation process of rock cracks because the whole process ends at only a few microseconds. One critical reason is that the time resolution of the high-speed camera only reached the degree of microseconds. But the process of rock fracture also ended at few microseconds. Therefore, clear images were difficult to obtain in rock fracture mechanics experiments. The ultrafast time-resolution method was widely used in other fields, such as spectroscopy and electron microscopy^20,21^. However, it has not been employed in rock mechanics experiments yet.

**Figure 1:**
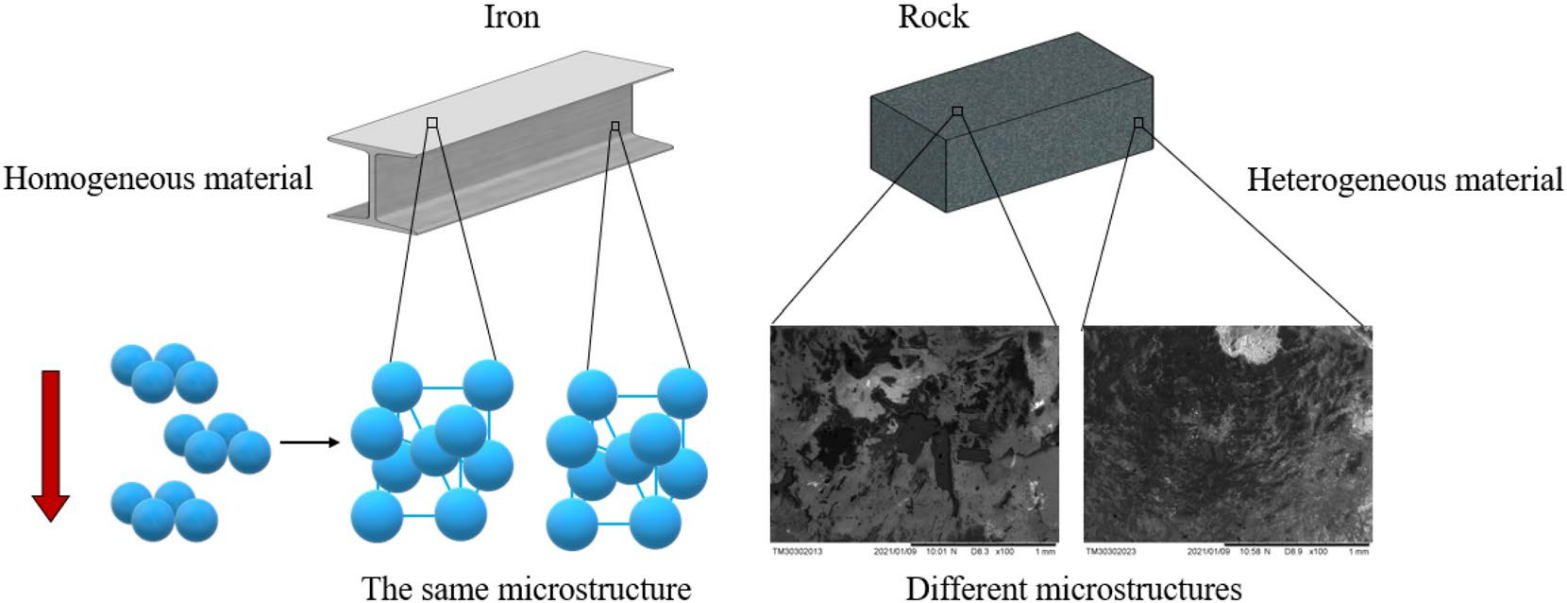
The microstructure of typical homogeneous material and heterogeneous material.

In the experiment of rock fracture, CT and acoustic emission are generally used to study the characteristics of crack propagation. Kou M. et al. studied the influences of confining pressures and internal fluid pressures on fracture behavior in rock-like materials subjected to both mechanical loads and internal hydraulic pressures by 3-D X-ray computed tomography combined with 3-D reconstruction techniques^22^. Wang Y. et al. performed multi-level cyclic compressive loading experiments on marble with different interbed orientations. And they revealed anisotropic fracture evolution characteristics using dynamic stress strain descriptions and post-test CT scanning technique^23^. Wang Y. et al. used real-time acoustic emission (AE) and post-test computed tomography (CT) scanning technologies to reveal the fracturing evolution and to further classify different crack types to aid in understanding dynamic fracturing^24^. Xue D. et al. re-constructed the model of the spatial correlation in fracture network by the acoustic emission (AE) signal cloud^25^. Zhao Y. et al. studied the failure characteristics of open flaws without any filling material by digital image correlation and acoustic emission monitoring technologies. And the test results are verified through numerical simulations^26^. Yang J. et al. explored the crack evolution among pre-existing flaws in rocks based on the acoustic emission (AE) source location results^27^. Some scholars use high-speed cameras to capture the whole process of rock crack propagation. Zhou Lei et al. studied dynamic fracture properties and found that the whole dynamic fracturing process of fractured rock under dynamic loads is composed of the cyclic process of crack initiation, high-speed crack propagation, slow deceleration, preventing crack propagation. Additionally, the period of crack obstruction was approximate the microsecond level^28^. Wong L. N. Y. and Einstein H. H. experimentally studied gypsum and Carrara marble specimens cracking and coalescence behavior at macroscopic and microscopic scales. And they used a high-speed video system to observe the cracking mechanisms^29,30^ precisely. With the aid of high-speed cameras, Zhou T. et al. studied 3D crack growth inside the transparent 3DP resin samples in real-time for the first time^31^. However, the time-resolution of the high-speed camera system used in previous research can only reach a microseconds degree. It is critical to improving the recording system's time resolution for further describing and studying the rock fracture characteristics. Additionally, as a typical heterogeneous material, the microstructure varies significantly at different locations. The microstructures of rock are very different from that of homogeneous materials such as steel, as shown in Fig. 1. Therefore, this makes the variation of fractures toughness of multipoint. However, relatively little work has been done to focus the change of fracture toughness at different positions.

Studying the fracture process of heterogeneous rock is of great help to establish rock fracture model, engineering stability, hydraulic fracturing, and other engineering applications. For example, effective rock fracture may improve the ore recovery ratio through effective separation of minerals along their grain boundaries in mineral processing^32–35^. Jialiang Liu et al. studied the crack evolution rules of hydraulic fracturing rock with different hardness^36^. By conducting a series of calculation analyses of the crack rheological fracture under different hydraulic pressures, Yanlin Zhao et al. found that wing crack propagation can be divided into two parts: the transient crack propagation at very high velocity and subcritical crack propagation at extreme low velocity^37^. Huang, R. Q et al. carried out experiments on pre-cracked sandstone to simulate failure of overhanging rock and verify the analytical solution to fracturing and failure of overhanging rocks^38^.

This paper proposed an innovative ultrafast time-resolution method based on a picosecond pulsed laser. Its lower degree of time resolution is 10^−11^ s. This method provides a new way to describe the whole process of rock fracture. Compared with high-speed photography in previous studies, the position of the rock crack tip will be determined at the accurate moment, rather than the overlying of images in the whole exposure time. Therefore, traditional time resolution method cannot meet the requirements of the experiment. The rock fracture rate is often about 300–700 m/s, and the material scale of the experimental samples in the laboratory experiment is on the order of 10^−2^ m. Therefore, the whole fracture process lasts only 10^−5^ s. In order to accurately describe the whole process of fracture, at least 10–100 images are required. At the same time, the time resolution of the recording method needs to be at least two orders of magnitude higher than the duration of the event to ensure the clarity of the image. Therefore, the time resolution of the measurement method is required to reach 10^−8^–10^−9^ s. However, due to the limitation of the camera sensor, the shutter time of the camera can reach 10^−7^ s at most. According to the above analysis, this time resolution must be forced to choose between the needs of capturing multiple fracture events and clear images. Then, we proposed a new method for determining rock fracture toughness during the crack propagation based on this method. The fracture toughness of multipoint on the rock crack propagation path can be determined by this method. Additionally, this new method emphasizes the effect of fracture toughness on crack propagation, which enables determining the fracture toughness along with the crack length and is closer to the original definition of fracture toughness.

## Experimental preparations and methods

### Experimental preparations

**Figure 2:**
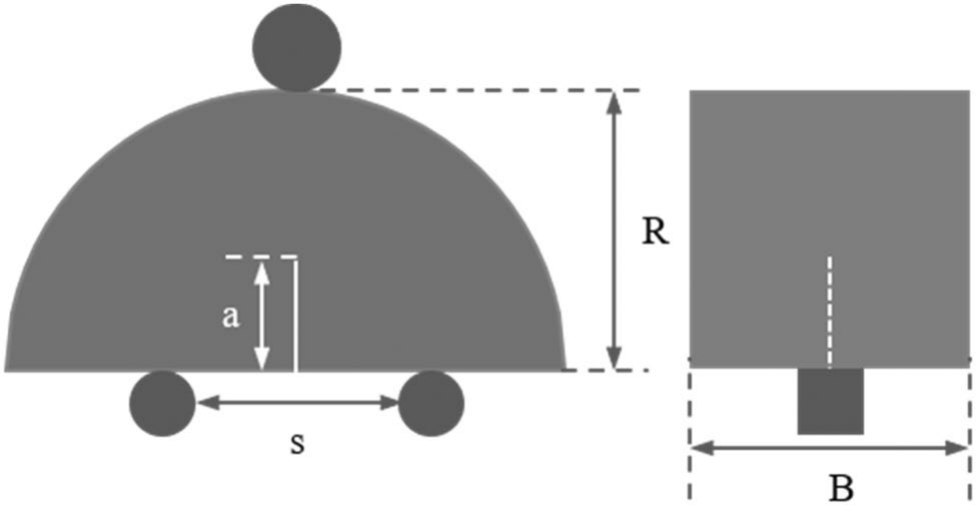
The schematic loading arrangement of tuff SCB specimen.

The SCB specimens were taken from tuff cores in Tibet. And their geometric dimensions are processed in strict accordance with the size range recommended by ISRM^39^. The specimens were pre-set with a crack of 0.5 mm wide and 10 mm long by wire-electrode cutting. The mechanical and geometric parameters of the specimens are shown in Table 1. The schematic loading arrangement of tuff SCB specimen used in this experiment is shown in Fig. 2.

**Table 1:** Mechanical and geometrical parameters of tuff specimen.

Uniaxial compressive strength	140.65 MPa
Elastic modulus	67.12 GPa
Poisson's ratio	0.192
Density	2.77 g/cm^3^
Radius (R)	50 mm
Thickness (B)	25 mm
Precast crack length (a)	10 mm
Distance between the two supporting cylindrical rollers (s)	25 mm

### Experimental method

**Figure 3:**
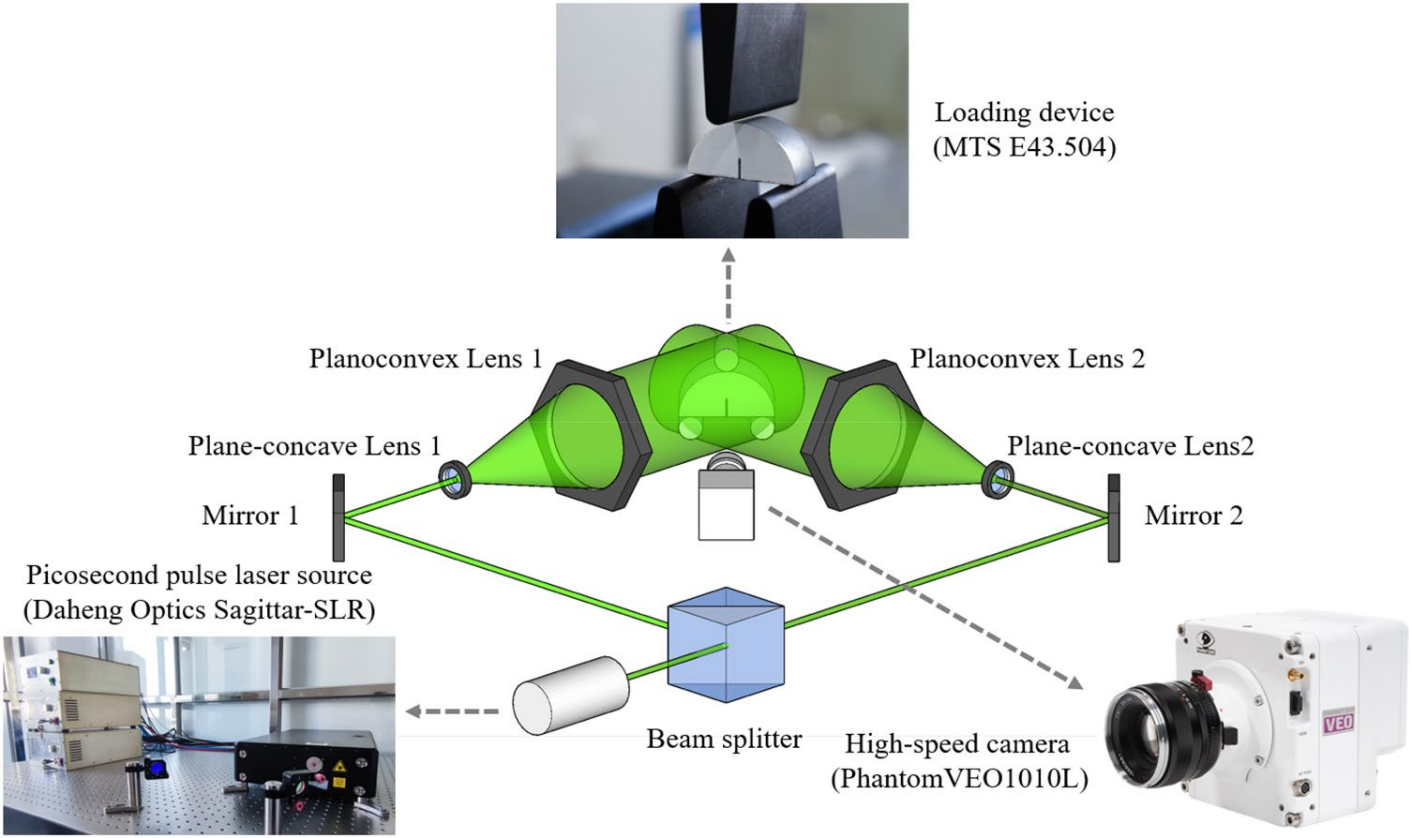
Experimental system setup.

The experimental system is shown in Fig. 3. This system consists of Picosecond pulsed laser source Sagittar-SLR made by Daheng Optics, loading device E45.504 made by MTS, spectroscope, mirror group, concave lens, convex lens, and high-speed camera. The pulsed laser source irradiates the tuff specimen through the spectroscope, reflector, concave lens, and convex lens, respectively. In order to capture the whole process of fracture with ultrafast time-resolution, the frame rate of the high-speed camera was set to 100 kfps. The wavelength of picosecond laser is 532 nm, the repetition frequency is 100 k/s, and the full width at half peak (FWHM) of laser is 15 ps.

**Figure 4:**
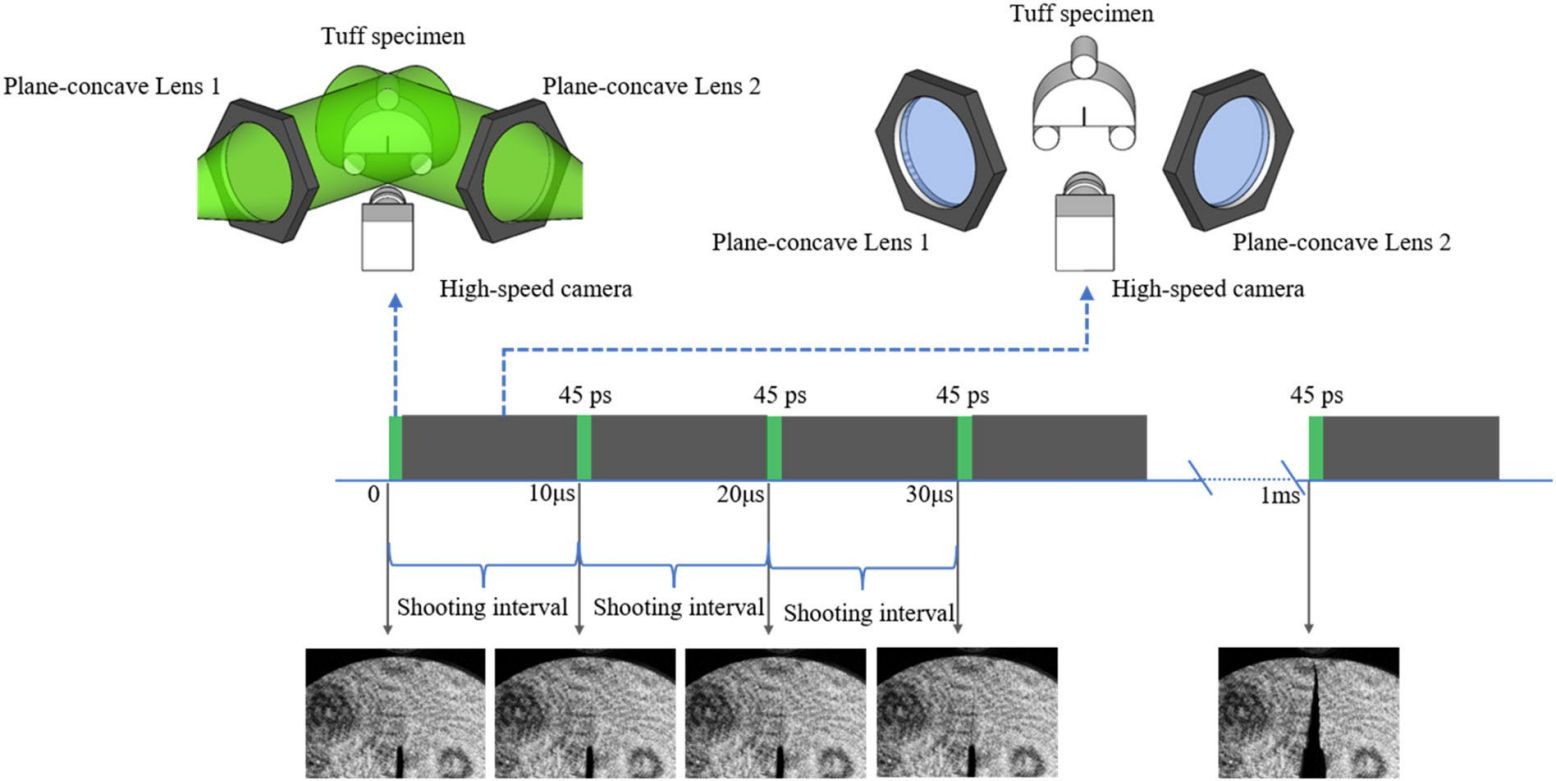
The schematic of the relationship between laser repetition rate and high-speed camera frame rate (the green part represents laser incidence).

The role of each component in the operation process of the experimental system in Fig. 3 is as followed. (1) The Picosecond pulsed laser source provided pulsed laser for shooting. (2) The beam splitter divided the laser into two beams of the same intensity. (3) The mirror1 and mirror2 adjusted the laser direction. (4) The plane-concave lens expanded the laser beam. (5) Finally, the Planoconvex lens adjusted the expanded laser to parallel light. (6) The loading device provided the force to break the specimens. (7) And the High-speed camera recorded the fracture processes of tuff specimens. The schematic of the relationship between laser repetition rate and high-speed camera frame rate is shown in Fig. 4.

The laser is repeated only once in one exposure interval of the high-speed camera, and the specimens were in the dark at the remaining time of exposure interval. The time resolution is high as 45 picoseconds because the exposure time of the system only depends on the FWHM of the pulsed laser. Actually, the FWHM of the pulsed laser was used as an “optical shutter.” While the frame rate of the high-speed camera only determines how many images were obtained during the fracture process.

### Experimental procedure

**Figure 5:**
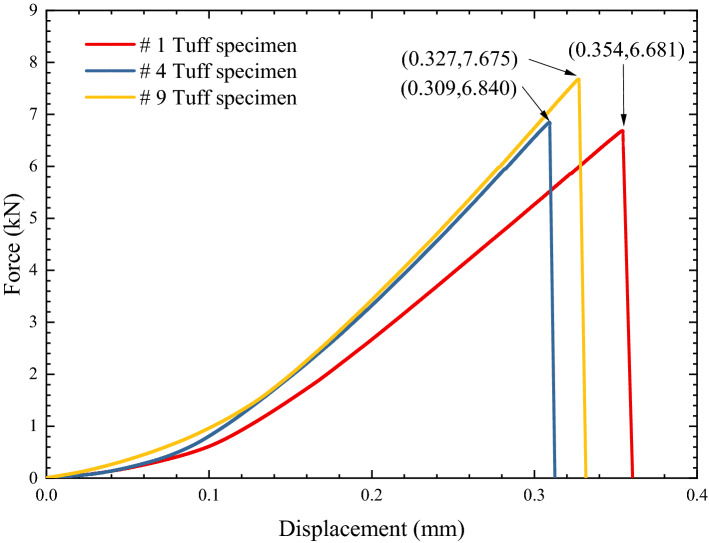
The force–displacement curve of tuff specimens.

1. The static mechanical parameters of rock were studied in this paper, which means no sudden change in the load during the loading process. Therefore, the specimens were loaded until fractured at 0.05 mm/min loading rate. And this loading rate is within the range of the ISRM suggested methods for rock testing. The force–displacement curve is shown in Fig. 5.
2. At the same time, the images were recorded by a high-speed camera. In this experiment, the distance and angle between the high-speed camera and the specimen were fixed. The original image was binarized after the experiment to track the crack tip more accurately.
3. The fracture rate can be determined by the crack length of each image and interval time between two images. Because the process is a linear-elastic brittle fracture, the load of specimen corresponding to each image can be obtained by interpolation according to the linear-elastic fracture theory.

## Experimental results and analysis

### Experimental results

**Figure 6:**
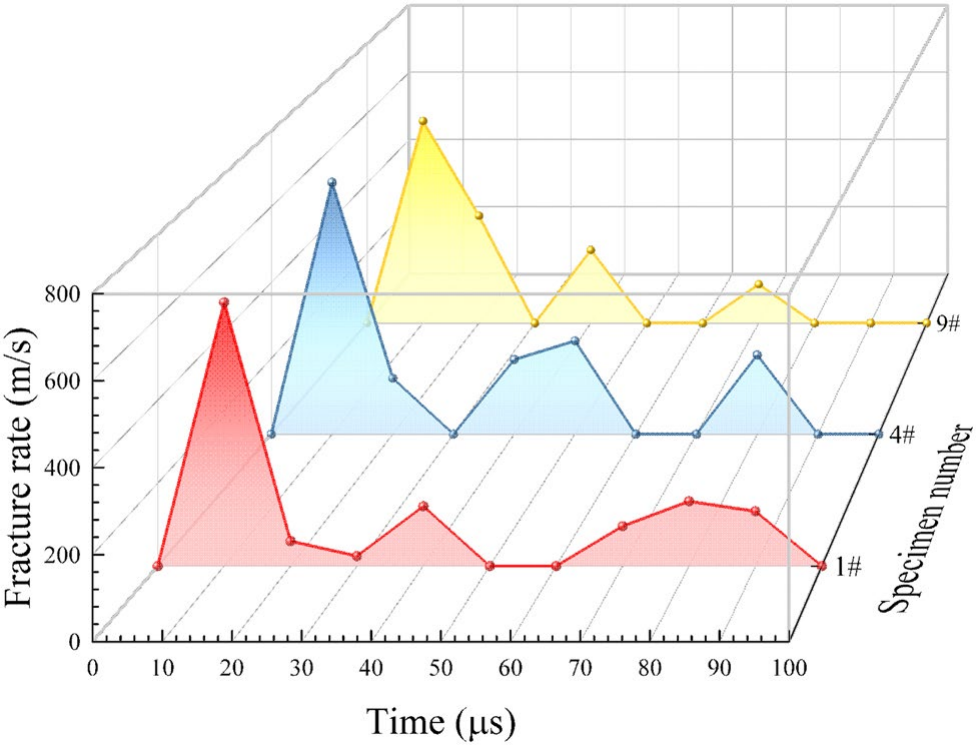
The fracture rate–time curve of tuff specimen during the whole process.

The fracture rate of the tuff specimens conducted in this experiment is shown in Fig. 6. To describe the experimental results clearly, the time before crack propagation was noted as 0 μs on the time axis. The crack propagation rates of the three specimens were 636 m/s, 663.9 m/s, and 578 m/s, respectively, in the first 10 μs. The crack propagation rates of the three samples decreased gradually in 10–30 μs, and the cycle of crack initiation, crack propagation and crack arrest appeared. Such cycles repeatedly appeared in the whole processes of the tuff specimens’ fractures. The crack propagation rates of the whole fractures processes were the mean propagation rates of multiple crack cycles. Because of its complex structure and inhomogeneity, the crack propagation rates of tuff specimens were not fixed but rather fluctuated wildly.

When the load reached the critical value, the load decreased rapidly, and the crack grew rapidly. This process is considered as an unstable propagation of microcracks. While, after the crack grew for a small distance, it can continue to grow only by increasing the load. Such a process is considered subcritical crack propagation. Obviously, the fracture process of the tuff specimen is a combination of multiple subcritical crack propagation and unstable propagation of microcracks. The crack propagation rates for the whole fracture process of three specimens were 11.19 m/s, 19.23 m/s, and 26.79 m/s, respectively. They were much lower than homogeneous materials with similar densities^40^.

**Figure 7:**
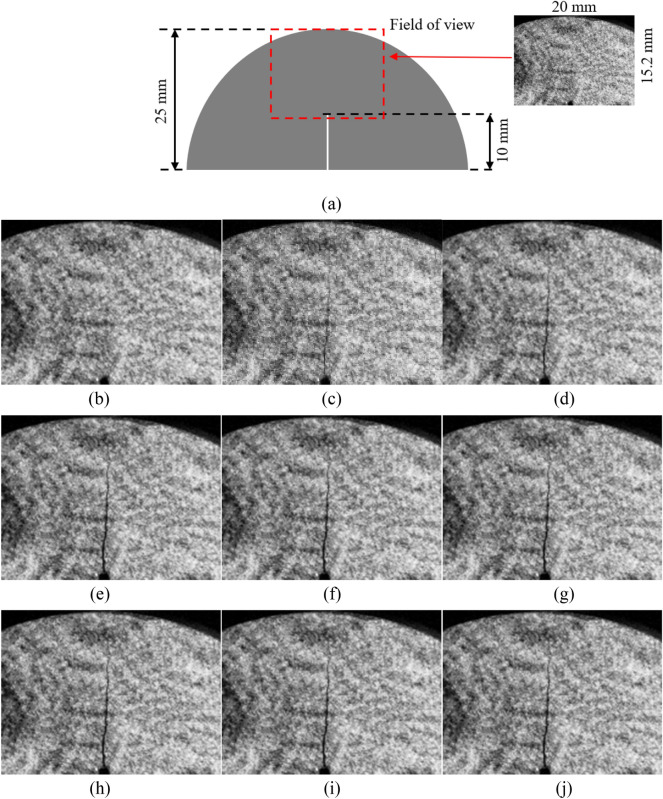
Crack propagation process of # 1 tuff specimen recorded in 20 μs intervals.

The crack propagation process of the # 1 specimen every 20 μs was shown in Fig. 7. The pulsed picosecond laser used in this study differs from the continuous light source widely used in the previous study. It lasted only 45 ps per exposure moment. The captured images correspond to brief moments rather than overlying the entire exposure time in previous high-speed photography techniques. This method avoids the blurring of traditional high-speed photography. Then a series of clear images of rock fracture can be obtained.

### A new physical model of the rock crack propagation

According to Irwin and Griffith's stress strength factor theory, the stress intensity factor at the fractured moment of the specimen is its fracture toughness. The stress intensity factor is a physical quantity related to load and geometry. However, fracture toughness is the inherent physical property of materials, which reflects the ability of materials to prevent crack propagation. It is independent of load and geometry. ISRM-Suggested method to determine the static fracture toughness of geotechnical materials only needs and geometry the peak load. While this method can only determine the static fracture toughness of the tip of the prefabricated crack. Geotechnical materials are heterogeneous, and the microstructure varies significantly at different positions. Therefore, their fracture toughness varies greatly at different positions. It is not enough to describe the fracture toughness of the whole material only by measuring the fracture toughness at the crack tip. And this method also fails to demonstrate fracture toughness's ability to prevent macroscopic cracks from propagating.

Based on the above experimental results, we proposed a new physical model to determine the static fracture toughness of geotechnical materials at multiple points on the crack propagation path. The fractures form of tuff specimens was not unstable propagation of microcracks. But multiple crack arrests occurred during the whole process. Because the fracture form of the sample in this study is the brittle linear elastic fracture. The ISRM-recommended method can still determine the stress intensity factors at multiple points on the crack propagation path. We can assume a virtual quasi-static loading process with servo loading device, and the servo loading device can only be loaded quasi-static in the loading process. The load decreased when the crack initiation occurred. Then the servo loading equipment was removed when it reached the crack arrest position. At this time, the energy release rate *G* and the expansion resistance *R* met the following relationship, and the crack arrested.

1 $$G\le R$$

2 $$\frac{\partial G}{\partial a}\le \frac{\partial R}{\partial a}$$

After crack arrest occurred, the specimen continued to be loaded. Until the subsequent fracture occurred, the stress release rate and fracture toughness met the following relationship:

3 $$G\ge R$$

4 $$\frac{\partial G}{\partial a}\ge \frac{\partial R}{\partial a}$$

**Figure 8:**
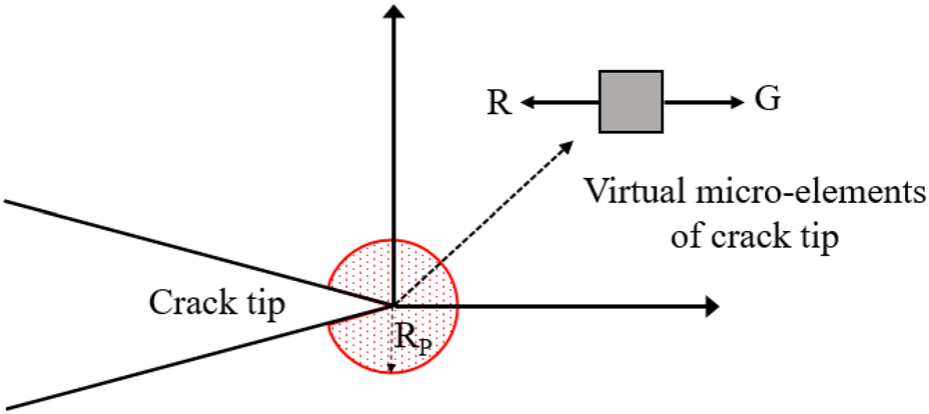
The schematic diagram of virtual micro element at crack tip (R_p_ is the radius of the plastic zone determined based on the von Mises yield criterion, R is fracture toughness, and G is stress intensity factor).

Due to the different compositions of mineral particles and structure, expansion resistance changed with the crack tip positions. And, energy release rate changes with stress. The dimensions of energy release rate and fracture toughness are $\frac{M}{{T}^{2}}$. In order to determine the fracture toughness of multiple points, as shown in Fig. 8, we first assume a virtual microelement at the crack tip, and its radius is determined by the von Mises yield criterion. In this study, the virtual element radius is set as 0.1 ‰ of the radius of the von Mises plastic yield zone. Then, the acceleration and deceleration of the virtual microelement can be used to represent the crack initiation and crack arrest. Figure 8 is a schematic diagram of the movement of the virtual microelement at the crack tip.

According to Newton's second law, the following formula can be obtained, where *V* is velocity, *t* is time, and *M* is mass.

5 $$B(G-R)=\frac{\partial V}{\partial t}dM$$

where *E* is elastic modulus

6 $$G=\frac{{K}_{I}^{2}}{E}$$

Under the condition of plane stress, the following formula can be obtained from von Mises yield criterion, where ${\sigma }_{y}$ is the maximal tensile strength.

7 $$dM=\frac{1}{2}\rho B{\int }_{0}^{2\pi }\frac{{K}_{I}^{4}}{4{\pi }^{2}{\sigma }_{y}^{4}}{\mathrm{cos}}^{4}\frac{\theta }{2}\left(1+{3\mathrm{sin}}^{2}\frac{\theta }{2}\right)d\theta $$

The relationship between ${K}_{IC}$ and $R$ is as follows:

8 $${K}_{IC}=\sqrt{\frac{R}{E}} $$

**Figure 9:**
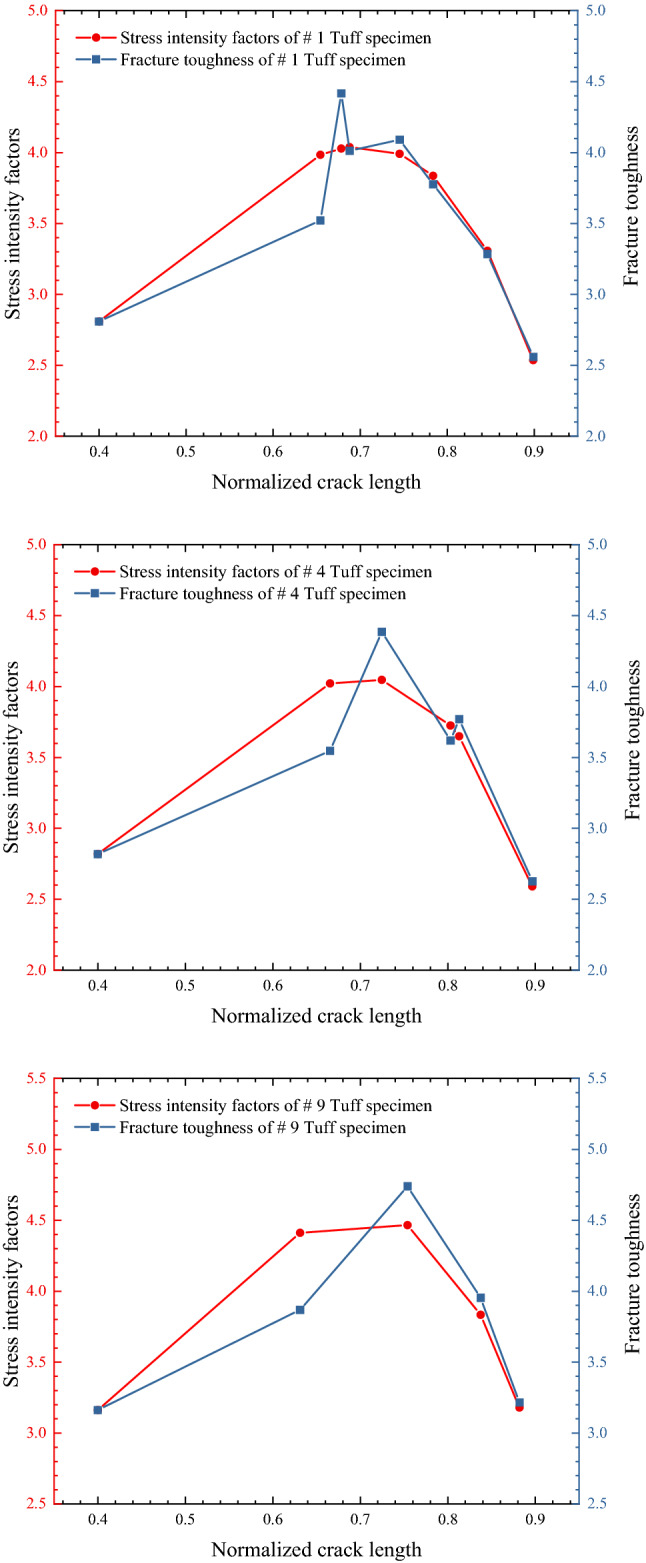
Fracture toughness and stress intensity factor at multiple points of tuff specimens.

The fracture toughness determined according to the above method is shown in Fig. 9. The deviation between fracture toughness ${K}_{IC}$ and stress intensity factor $K$ was the largest at the initial crack propagation stage. Moreover, the acceleration of virtual microelements changed significantly, reflecting the significant change of crack propagation rate. In the later stage of crack propagation, it gradually approached the stress intensity factor, and the variation of virtual micro acceleration was slight, reflecting that the crack propagation rate changed minor or crack arrest occurred.

## Summary and conclusion

We captured the whole process of tuff crack propagation through this method in the time-resolution of 45 picoseconds. It is found that the fracture process of tuff is different from that of previous studies and other homogeneous brittle materials. The process of tuff crack propagation was a repeated combination of crack initiation, accelerated propagation, and crack arrest. The process of accelerating propagation with decreasing load is considered to be crack unstable propagation of microcracks. As the aggregation of various kinds of mineral particles, their mechanical parameters vary greatly. This leads to the different difficulties of crack propagation at different positions on the crack propagation path. Furthermore, microcracks will connect in the process of rock fracture, and the inclinations of these microfractures are different at different positions. The above results in a significant variation of rock crack propagation rate.

We assumed a virtual microelement at the crack tip to describe the difficulty degrees of crack propagation in tuff along the crack propagation path. The relationship between energy release rate *G* and expansion resistance *R* is obtained through the acceleration and deceleration of virtual microelements. Then the fracture toughness at multi-position along the crack propagation path was determined. The results showed that the difference between fracture toughness and stress intensity factor is significant at the initial crack propagation stage, which corresponded to the initial stage of crack propagation and the stage of rapid crack propagation. In the later stage, the fracture toughness is approximately the same as the stress intensity factor, which corresponds to the process of stable crack propagation or stopping fracture. Through this method, we also determined the variation of multi-point fracture toughness of tuff specimen with strong heterogeneity during crack propagation.

In the previous study on the fracture toughness of brittle materials, the critical stress intensity factor of the material at the fractured moment is used as the material's fracture toughness. This method is reasonable and accurate inhomogeneous and isotropic materials. However, rock has strong heterogeneity and complex structure, it is obviously unreasonable to use the critical stress intensity factor of one point as the fracture toughness of the whole specimen. Therefore, this method provides a new angle to determine the multi-point fracture toughness of heterogeneous materials such as rock. It will contribute to studying brittle material fracture of complex joints such as shale in the future.

The fracture toughness is related to the acceleration and deceleration of virtual microelements at the crack tip. This also reflects the physical meaning of fracture toughness. Because of its ultrafast time-resolution, this method is also helpful for experiments with high strain rates, such as Hopkinson compression bar experiment, explosion load experiment, and pendulum impact experiment.
